# Effect of *Allium* spices (garlic and onion) on the bioaccessibility of iron from *Moringa oleifera* leaves

**DOI:** 10.1002/fsn3.3913

**Published:** 2023-12-22

**Authors:** Saliou Mawouma, Florence Doudou Walko, Jude Mbyeya, Souaibou Hamidou Yaya, Emmanuel Awoudamkine, Carl Moses Mbofung Funtong

**Affiliations:** ^1^ Department of Biological Sciences, Faculty of Science University of Maroua Maroua Cameroon; ^2^ Department of Food Science and Nutrition, National School of Agro‐industrial Sciences University of Ngaoundere Ngaoundere Cameroon

**Keywords:** *Allium cepa*, *Allium sativum*, bioaccessibility, iron, *Moringa oleifera* leaves

## Abstract

The aim of this study was to investigate the effect of garlic and onion, two *Allium* spices rich in sulfur compounds, on the bioaccessibility of iron from *Moringa oleifera* leaves. We first quantified anti‐nutritional factors in various cooked mixtures of *Moringa oleifera* leaves and spices, with increasing level of incorporation of garlic or onion. We then assessed the iron bioaccessibility of the various mixtures using a simulated in vitro digestion method. Finally, we studied the speciation of bioaccessible iron. Total phenols contents ranging from 801.44 to 903.07 and from 869.78 to 990.72 mg/100 g of dry matter in garlic and onion mixtures, respectively, increased (*p* < .05) with the level of incorporation of spices. Phytates contents followed the same tendency with values ranging from 1.84 to 2.12 and from 1.75 to 2.02 mg/100 g of dry matter in garlic and onion mixtures, respectively. Although the presence of garlic and onion significantly reduced (*p* < .05) the total iron content of the mixtures (11.56–11.96 mg/100 g of dry matter), we noticed that bioaccessible iron was significantly higher (*p* < .05) in spiced mixtures (36.35%–48.40%) compared to the control (23.28%), with the greatest amount found in the mixture containing 10 g of onion. The predominant specie of bioaccessible iron was organic iron, whose amounts in the spiced mixtures (0.59–0.69 mg/L) were all significantly higher (*p* < .05) than in the control (0.32 mg/L). Globally, the presence of spices produced no significant variation (*p* > .05) in amounts of ferrous iron, the major inorganic specie of bioaccessible iron. The use of garlic and onion as ingredients could help improving the iron status of populations consuming iron‐rich leafy vegetables.

## Graphical abstract

The bioaccessibility of iron from *Moringa oleifera* leaves in the presence of garlic and onion, two spices rich in sulfur compounds, was investigated. Although the incorporation of spices increased anti‐nutritional factors contents and generated disadvantageous phytates/iron molar ratios, there was a net improvement in iron bioaccessibility. Promoting the use of garlic and onion as ingredients in the preparation of iron‐rich leafy vegetables could be a simple and efficient mean to combat iron deficiency.

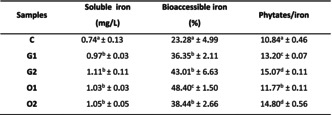

## 1 INTRODUCTION

Anemia, which is characterized by insufficient levels of erythrocytes or hemoglobin in the blood, affects 25% of the world's population, mainly found in developing countries (Balarajan et al., 2011). A survey conducted by Nchanji et al. (2020) revealed that the prevalence of anemia among Cameroonian children is estimated to be 64% in the Far North region, with children aged 6–59 months being more affected. Women aged 15–49 were also found to suffer more from anemia in this region.

In adults, iron deficiency leads to reduced physical capacity and productivity (Blakstad et al., 2020). Severe anemia is responsible for 20% of maternal deaths (Teshome et al., 2020) and increases the risk of fetal morbidity and mortality, as well as prematurity and low birth weight (Rocha et al., 2021). Furthermore, in anemic children, growth, physical performance, and immune defenses are impaired, increasing infectious morbidity (Cappellini et al., 2020).

The main causes of iron deficiency in underdeveloped countries are poverty and diets based on plant foods, which contain high levels of anti‐nutritional factors (Dykes & Rooney, 2007; Neugart et al., 2017). Anti‐nutritional factors form non‐absorbable chelates with non‐heme iron, reducing its absorption in the gastrointestinal tract (Rousseau et al., 2020). In addition, animal proteins (meat, fish, poultry) containing better‐absorbed heme iron are not accessible to all social classes due to their high cost (Milman, 2020).

Programs implemented worldwide to combat micronutrient deficiencies have integrated various approaches, including supplementation, fortification, and dietary diversification (Dubock, 2017). These solutions generally focus only on increasing micronutrient levels in foods, without taking into consideration their bioaccessibility. Bioaccessibility is defined as the fraction of a nutrient released from the food matrix into the gastrointestinal tract and available for absorption (Wu & Chen, 2021). The high cost of micronutrients supplements and the risk of toxicity are additional limitations of supplementation (Gibson & Hötz, 2001; Welch, 2002).

One of the dietary solutions promoted by FAO (Food and Agriculture Organization) and WHO (World Health Organization) to combat iron deficiency in poor countries is improved culinary techniques. Acidulation of traditional dishes has shown efficiency in improving iron bioaccessibility (Mawouma et al., 2017; Musa & Ogbadoyi, 2012; Schönfeldt & Pretorius, 2011). However, acidic dishes are not always appreciated by all individuals in the population and may not be suitable for people suffering from gastric ulcers. The use of spices from the *Allium* genus (onion and garlic) improved the bioaccessibility of iron and zinc from cereals and pulses (Gautam et al., 2010). These spices are rich in sulfur compounds that could form soluble chelates with ionic iron and improve its intestinal absorption, even in the presence of anti‐nutritional factors such as phytates and polyphenols (Greger & Mulvaney, 1985). Aside from cereals and pulses, leafy vegetables are other important sources of dietary iron. Previous studies reported *Moringa oleifera* leaves to be particularly rich in iron (Arora & Arora, 2021). *Allium sp* spices are therefore of great interest in the dietary approach of combatting iron deficiency, since they could be used as ingredient in the preparation of *Moringa oleifera* leaves mostly eaten in the cooked form in many regions of the globe.

The objective of this study was to investigate the effect of *Allium sp* spices on the bioaccessibility of iron from *Moringa oleifera* leaves.

## 2 MATERIALS AND METHODS

### 2.1 Biological material

Fresh and mature *Moringa oleifera* leaves were harvested from the same tree in a home garden. They were then washed under running tap water to remove dust, and the excess water was drained off. Onion (A*llium cepa*) and garlic (*Allium sativum*) bulbs were purchased at a market in the town of Maroua (Far North region of Cameroon).

### 2.2 Sample preparation

The various samples were formulated as shown in Table 1. The proportions of leaves and spices were chosen on the basis of common culinary techniques for preparing *Moringa oleifera* leaves in the Far North region of Cameroon (Mawouma & Mbofung, 2014). To investigate a possible dose–effect, two levels of incorporation of each spice were set.

**TABLE 1:** Description of samples.

Samples	FMO (g)	Garlic (g)	Onion (g)	Deionized water (g)
C	50	–	–	150
G1	50	5	–	150
G2	50	10	–	150
O1	50	–	10	150
O1	50	–	20	150

Abbreviations: C, control mixture; FMO, fresh *Moringa oleifera* leaves; G, Garlic mixtures; O, Onion mixtures.

*Moringa oleifera* leaves, onion, and garlic were cut into small pieces, mixed with deionized water according to Table 1 and pulverized using a domestic blender for 30 s. The resulting mixtures were transferred to plastic bags, and then cooked in a boiling water bath for 30 min. Each cooked sample was divided into two aliquots: one aliquot was oven‐dried (BINDER GmbH) at 105°C for 24 h to determine water content and total iron content, and the another aliquot was stored below 0°C for further analyses.

### 2.3 Chemical analysis

#### 2.3.1 Determination of anti‐nutritional factors contents

Total phenol contents were determined using the method of Shi and Le Maguer (2003). Each sample (0.5 g) was mixed with 10 mL of HCl‐acidified methanol (70%) in a test tube. The whole was vortexed for 5 min and left to stand for 24 h at room temperature. The extract was obtained after filtration through Whatman N°1 filter paper. In a test tube, 0.1 mL of extract was mixed with 7.9 mL of distilled water, and 0.5 mL of Folin–Ciocalteu reagent. After 10 min, 1.5 mL of 20% sodium carbonate was added and the whole was homogenized and left to stand for 1 h in the dark. The absorbance was read at 765 nm and a standard curve (0.1–0.5 mg/mL of gallic acid, *R* ^2^ = .99) was used to calculate the concentration of total phenols in the samples.

Phytates were determined using the method of Bhandari and Kawabata (2006). In a centrifuge tube, 0.5 g of each sample was introduced and mixed with 10 mL of HCl 2.4%. The mixture was vortexed for 5 s, and the phytates were extracted for 1 h at room temperature under regular agitation. After centrifugation at 3500 rpm for 30 min, 1 mL of each supernatant, 1 mL of distilled water and 1 mL of Wade reagent (0.03% FeCl_3_.6H_2_O + 0.3% sulfosalicylic acid) were introduced into a test tube, then vortexed for a few seconds and centrifuged at 3500 rpm for 10 min. Absorbance was read at 500 nm. A standard curve (0–40 mg/mL of phytic acid, *R* ^2^ = .96) was used to calculate phytates concentrations in the samples.

#### 2.3.2 Determination of total iron content

Total iron content in dehydrated samples was determined by colorimetry using potassium thiocyanate (KSCN) according to the method described by Pauwels et al. (1992). Total iron in a mineralized and solubilized sample is oxidized by hydrogen peroxide in acidic conditions to Fe(III) ions. Fe(III) ions are revealed by a solution of potassium thiocyanate through the formation of a red complex which has a maximum absorbance of 420 nm. Practically, dehydrated samples were calcined in a muffle furnace at 450°C, and then digested in 1 mol/L nitric acid. The digested solutions were centrifuged at 4000 rpm for 10 min. Five milliliters (5 mL) of supernatant was introduced into a test tube, and then few drops of hydrogen peroxide and 1 mL of KSCN were added. After 10 min of incubation, absorbance was read at 420 nm. A standard curve (0–10 ppm of Fe(III), *R* ^2^ = .99) was used to calculate the total iron concentrations in the samples.

#### 2.3.3 Determination of iron–polyphenol complex content

Iron–polyphenol complex content was determined directly in acidified methanol extracts using the method of McGee and Diosady (2018). Polyphenols can chelate iron to form a complex that is not absorbed in the gastrointestinal tract, with maximum absorbance at 555 nm in acidic conditions. A standard curve (0–0.5 mmol/L of Fe/gallic acid solutions, *R* ^2^ = .88) was used to calculate the iron–polyphenol complex contents in extracts.

#### 2.3.4 Determination of iron bioaccessibility

##### Predictive method: Calculation of phytates/iron molar ratios in mixtures

The molar ratio between phytates and iron was calculated by dividing the number of moles of phytates by the number of moles of iron.

##### In vitro digestion method

In vitro digestion of mixtures was conducted using the method described by da Silva et al. (2017). Simulated gastric juice was prepared by dissolving 0.48 g of pepsin (Sigma P7125) in 10 mL of HCl (0.1 mol/L). Simulated intestinal juice was prepared by dissolving 0.06 g of pancreatin (Sigma P7545) and 0.375 g of bile extract (Sigma B8631) in 15 mL NaHCO_3_ (0.1 mol/L).

###### Gastric digestion

In a glassware previously rinsed with deionized water, 5 g of sample was dissolved in 10 mL of deionized water. The pH was adjusted to 2 with a solution of concentrated HCl (2.4%), and then the mixture was incubated at 37°C for 10 min before adding 0.1 mL of pepsin solution. The whole mixture was incubated at 37°C for 2 h under regular gentle agitation.

###### Intestinal digestion

The pH of the gastric digestate was adjusted to 5 with NaHCO_3_ (1 mol/L), then 0.64 mL of pancreatin–bile extract solution was added. The mixture was incubated for 2 h at 37°C under regular gentle agitation. The digestates were cooled in an ice bath for 10 min to inactivate enzymes. The pH was then adjusted to 7.2 with NaOH (0.1 mol/L) and centrifuged at 3500 rpm for 5 min. The intestinal digestate was then filtered through Whatman N°1 filter paper. Filtrates in which soluble iron had been released during digestion were stored below 0°C for soluble iron quantification as described above for total iron.

The percentage of bioaccessible iron was determined according to the following formula:

(1) $$\text{Bioaccessibility}\;\left(\%\right)=\frac{\text{Quantity of mineral in the filtrate}}{\text{Quantity of mineral in the sample}}\;x\;100$$

#### 2.3.5 Speciation of bioaccessible iron

The iron speciation study was carried out using the method described by da Silva et al. (2017). This method uses the principle that ferric iron Fe(III) in the presence of hydroxylamine hydrochloride is reduced to ferrous iron Fe(II) which forms a stable colored complex (red‐orange) with 1‐10‐phenanthroline having a maximal absorbance at 510 nm.

Two millimeters (2 mL) of filtrate from in vitro digestion were mixed with 0.16 mL sodium nitrite (0.39%), 1 mL of protein precipitating solution (5 g TCA + 5 mL of concentrated HCl for 50 mL of solution), and 6.84 mL of deionized water. The mixture was incubated at 100°C for 10 min and then centrifuged at 3500 rpm for 15 min.

##### Quantification of inorganic iron

Two milliliters (2 mL) of supernatant were introduced into a test tube. Then, 1 mL of hydroxylamine hydrochloride (1%) and 1 mL of 1,10‐phenanthroline (0.25%) were added. The homogenized mixture was allowed to stand for 15 min, and then the absorbance was read at 510 nm. A standard curve (0–5 mg/L of FeSO_4_ solutions, *R* ^2^ = .99) was used to calculate the concentrations of total Fe(II) ions, corresponding to the total inorganic iron ions in the samples.

##### Quantification of ferrous iron Fe(II)

Two milliliters (2 mL) of supernatant were introduced in a test tube. Then, 1 mL of 1,10‐phenanthroline (0.25%) was added. The homogenized mixture was left to stand for 15 min, and then the absorbance was read at 510 nm. A standard curve (0–5 mg/L of FeSO_4_ solutions, *R* ^2^ = .99) was used to calculate the concentration of Fe(II) ions in the samples.

##### Determination of ferric iron Fe(III) content

Ferric iron Fe(III) content was determined by deductive calculation according to the following formula:

(2) Ferric iron=Inorganic iron−Ferrous iron

##### Determination of organic iron content

Organic iron content was deduced using the following formula:

(3) Organic iron=Soluble iron−Inorganic iron

### 2.4 Statistical analysis

Results were reported as mean ± SD. Data were subjected to analysis of variance (ANOVA) using IBM SPSS Statistics version 20.0 software. Differences between means were analyzed by Duncan's multiple range test with a significance level of *p* < .05.

## 3 RESULTS AND DISCUSSION

### 3.1 Anti‐nutritional factors contents in mixtures

Table 2 shows the total phenols and phytates contents of the mixtures. Total phenol contents ranged from 801.44 to 903.07 mg/100 g DM for garlic mixtures and from 869.78 to 990.72 mg/100 g DM for onion mixtures. It can be seen that these contents increased (*p* < .05) with the quantity of incorporated spices. These amounts are significantly higher (*p* < .05) than that of the control. This could be explained by the high phenol content of *Allium* spices (Fernández‐Bedmar et al., 2019). A recent study conducted by Khadija and Marwa (2021) revealed that garlic had a high content of total polyphenols (358 mg EAG/g), much higher than *Moringa oleifera* leaves (236.5 mg EAG/g) (Belhi et al., 2018). The same study also revealed that onion also had a high total polyphenol content (276 mg EAG/g), compared to *Moringa oleifera* leaves.

**TABLE 2:** Anti‐nutritional factors contents in mixtures.

Samples	Total phenols (mg/100 g DM)	Phytates (mg/100 g DM)
C	797.88^a^ ± 12.15	1.89^b^ ± 0.07
G1	801.44^a^ ± 20.68	1.84^b^ ± 0.02
G2	903.07^b^ ± 104.62	2.12^d^ ± 0.01
O1	869.78^a^ ± 47.40	1.75^a^ ± 0.01
O2	990.72^b^ ± 67.95	2.02^c^ ± 0.05

*Note*: Mean values in the same column with different superscript letters are significantly different (*p* < .05). G1: Mixture with garlic (5 g); G2: Mixture with garlic (10 g); O1: Mixture with onion (10 g); O2: Mixture with onion (20 g).

Abbreviations: C, Control mixture; DM, Dry matter.

Phytates levels ranged from 1.84 to 2.12 mg/100 g DM for garlic mixtures and from 1.75 to 2.02 mg/100 g DM for onion mixtures. These valies increased with the level of incorporated spices and were higher (*p* < .05) than that of the control, especially when the quantity of spices added was doubled. This increase could be due to the high phytates content of the spices (Cuendet, 1999; Macheix et al., 2005; Vermerris, 2006) which would have increased the phytates content of the mixtures. The results obtained showed that garlic mixtures are richer in phytates than onion mixtures. A study conducted by Salawu et al. (2021) revealed that garlic is richer in phytates (1.79 mg/kg) than onion (1.45 mg/kg).

Polyphenols and phytates form insoluble complexes with non‐heme iron, preventing its absorption from the gastrointestinal tract (Hurrell, 2003). The higher amounts of these compounds in spiced mixtures could compromise the bioaccessibility of their iron content. However, the contribution of sulfur compounds from garlic and onion could compete with the inhibitory effect of polyphenols and phytates.

### 3.2 Total iron and iron–polyphenol complex content of mixtures

Table 3 shows the total iron and iron–polyphenol complex contents of the various mixtures. The total iron content of the spiced mixtures was significantly lower (*p* < .05) than that of the control. This observation could be explained by the low iron content of spices (Akinwande & Olatunde, 2015) compared to *Moringa oleifera* leaves (Broin, 2005). A comparison of the values for garlic and onion mixtures shows that the latter are richer in iron.

**TABLE 3:** Total iron and iron–polyphenol complex contents of mixtures.

Samples	Total iron (mg/100 g DM)	Iron–polyphénols (mmol/L)
C	14.94^d^ ± 0.01	0.39^a^ ± 0.06
G1	11.96^b^ ± 0.17	0.41^a^ ± 0.11
G2	11.75^b^ ± 0.16	0.78^b^ ± 0.08
O1	12.56^c^ ± 0.12	0.38^a^ ± 0.16
O2	11.58^a^ ± 0.02	0.26^a^ ± 0.03

*Note*: Mean values in the same column with different superscript letters are significantly different (*p* < .05). G1: Mixture with garlic (5 g); G2: Mixture with garlic (10 g); O1: Mixture with onion (10 g); O2: Mixture with onion (20 g).

Abbreviations: C, Control mixture; DM, Dry matter.

The iron–polyphenol complex contents of the garlic mixtures increased with the addition of this spice and were significantly higher (*p* < .05) than that of the control. This can be explained by garlic's contribution to polyphenols (Liu et al., 2018). By doubling the amount of spice, the iron–polyphenol complex content was significantly higher (*p* < .05) than that of the control. There was no significant difference between the iron–polyphenol complex content of the onion mixtures and the control. This suggests a specific chemical environment in onion mixtures which could prevent the formation of iron–polyphenol complex despite the additional quantity of phenols brought by onion. One could hypothesize that the phenolic compound founds in onion could be less favorable to the formation of iron–polyphenol complex.

### 3.3 Iron bioaccessibility

Table 4 shows the soluble iron and bioaccessible iron contents, as well as phytates/iron molar ratios of the different mixtures. The soluble iron contents of digestates of spiced mixtures were significantly higher (*p* < .05) than that of the control. Sulfur compounds brought by spices could have favored the solubilization of a fraction of the iron pool of spiced mixtures. It is unlikely that soluble iron increased in spiced mixtures digestates because of the soluble iron contribution of spices. Indeed, *Allium* spices are less concentrated in iron compared to Moringa leaves and were minor constituents of the spiced mixtures. So the contribution of spices to soluble iron could be negligible. Also, the intrinsic anti‐nutritional factors found in spices could form insoluble chelates with iron, further reducing the contribution of spices to soluble iron in spiced mixtures.

**TABLE 4:** Bioaccessibility of iron content in mixtures.

Samples	Soluble iron (mg/L)	Bioaccessible iron (%)	Phytates/iron
C	0.74^a^ ± 0.13	23.28^a^ ± 4.99	10.84^a^ ± 0.46
G1	0.97^b^ ± 0.03	36.35^b^ ± 2.11	13.20^c^ ± 0.07
G2	1.11^b^ ± 0.11	43.01^b^ ± 6.63	15.07^d^ ± 0.11
O1	1.03^b^ ± 0.03	48.40^c^ ± 1.50	11.77^b^ ± 0.11
O2	1.05^b^ ± 0.05	38.44^b^ ± 2.66	14.80^d^ ± 0.56

*Note*: Mean values in the same column with different superscript letters are significantly different (*p* < .05). G1: Mixture with garlic (5 g); G2: Mixture with garlic (10 g); O1: Mixture with onion (10 g); O2: Mixture with onion (20 g).

Abbreviation: C, Control mixture.

The levels of bioaccessible iron in the digestates of spiced mixtures were significantly higher (*p* < .05) than that of the control. This result corroborates with the increase in soluble iron content in the digestates of spiced mixtures. Sulfur compounds brought by *Allium* spices could have considerably enhanced iron bioaccessibility. According to a study published in 2010, there was an increase in iron bioaccessibility in different cereals and pulses spiced with garlic (0.25 and 0.5 g/10 g of cereals or pulse) and onion (1.5 and 3 g/10 g of cereals or pulses). This study showed that the bioaccessibility of iron content of sorghum ranged from 13.9% to 37.6% and from 27.2% to 65.9% in the presence of garlic and onion, respectively (Gautam et al., 2010).

The spiced mixtures have phytates/iron molar ratios ranging from 11.77 to 15.07. These values are significantly higher (*p* < .05) than that of the control. This may be explained by the fact that the incorporated spices were rich in phytates. It was also observed that by doubling the quantities of garlic and onion, phytates/iron ratios increased significantly (*p* < .05). The mechanism by which phytates inhibit mineral absorption is based on the formation of insoluble phytate–mineral complexes in the gastrointestinal tract (Gibson et al., 2010; Kumar et al., 2010; Lonnerdal, 2010; Lopez et al., 2002). The molar ratios obtained in our study are all within the limits of a low iron bioaccessibility. The work of Lestienne et al. (2005) revealed that iron uptake decreased for phytates/iron ratios above 14. It is important to point out that the low bioaccessibility of iron implied by these molar ratios should be viewed with great caution. Indeed, the activating effect of the iron solubility of sulfur compounds in the studied mixtures would have supplanted the inhibiting effect of phytates.

### 3.4 Speciation of bioaccessible iron

Table 5 shows the amounts of inorganic iron, organic iron, Fe(II), and Fe(III) in digestates of the different mixtures. The organic iron contents of the spiced mixtures ranges from 0.59 to 0.69 mg/L and were significantly higher (*p* < .05) than that of the control. Generally speaking, the incorporation of spices favored the formation of the organic specie of bioaccessible iron. No significant difference (*p* > .05) was observed according to the type of spice (garlic or onion) or the level of incorporation of each spice. Organic iron is a soluble complex made of iron linked to an organic compound. The observed result suggests that sulfur compounds could have increased bioaccessible iron by chelating this metal.

**TABLE 5:** Speciation of bioaccessible iron.

Samples	Soluble iron (mg/L)	Inorganic iron (mg/L)	Organic iron (mg/L)	Fe(II) (mg/L)	Fe(III) (mg/L)
C	0.74^a^ ± 0.13	0.51^c^ ± 0.03	0.32^a^ ± 0.24	0.33^b^ ± 0.00	0.18^b^ ± 0.03
G1	0.97^b^ ± 0.03	0.37^a^ ± 0.00	0.59^b^ ± 0.02	0. 26^a^ ± 0.03	0.11^a^ ± 0.02
G2	1.11^b^ ± 0.11	0.41^a^ ± 0.01	0.69^b^ ± 0.11	0.30^b^ ± 0.02	0.11^a^ ± 0.02
O1	1.03^b^ ± 0.03	0.39^a^ ± 0.00	0.63^b^ ± 0.03	0.30^b^ ± 0.01	0.09^a^ ± 0.01
O2	1.05^b^ ± 0.05	0.45^b^ ± 0.04	0.59^b^ ± 0.08	0.30^b^ ± 0.03	0.14^b^ ± 0.03

*Note*: Mean values in the same column with different superscript letters are significantly different (*p* < .05). A1: Mixture with garlic (5 g); A2: Mixture with garlic (10 g); O1: Mixture with onion (10 g); O2: Mixture with onion (20 g).

As far as inorganic iron is concerned, the ionic ferrous form was more abundant than the ferric form. Globally, the incorporation of spices into Moringa leaves produced no significant variation in the levels of ferrous iron, the predominant inorganic form of bioaccessible iron. This result seems to confirm that iron complexation appears to be the main mechanism by which sulfur compounds promote iron bioaccessibility.

## 4 CONCLUSION

The aim of this research was to investigate the effect of garlic and onion, two spices of the *Allium* genus rich in sulfur compounds, on the bioaccessibility of iron from *Moringa oleifera* leaves. Although the incorporation of spices increased anti‐nutritional factors' contents, reduced the total iron contents, and generated disadvantageous phytates/iron molar ratios, there was a net improvement in iron bioaccessibility. Iron chelation by sulfur compound resulting in the formation of soluble complexes is a possible mechanism of this improvement. The use of garlic and onion as ingredients in the preparation of iron‐rich leafy vegetables could be a simple means to combat iron deficiency.

## AUTHOR CONTRIBUTIONS

**Saliou Mawouma:** Conceptualization (equal); data curation (equal); formal analysis (equal); investigation (equal); methodology (equal); resources (equal); software (equal); writing – review and editing (equal). **Florence Doudou Walko:** Conceptualization (equal); formal analysis (equal); investigation (equal); methodology (equal); resources (equal); writing – original draft (equal). **Jude Mbyeya:** Formal analysis (equal); investigation (equal); methodology (equal). **Souaibou Hamidou Yaya:** Formal analysis (equal); investigation (equal); methodology (equal). **Emmanuel Awoudamkine:** Visualization (equal); writing – original draft (equal); writing – review and editing (equal). **Carl Moses Mbofung Funtong:** Conceptualization (equal); supervision (equal); validation (equal); visualization (equal).

## FUNDING INFORMATION

The authors did not receive support from any organization for the submitted work.

## CONFLICT OF INTEREST STATEMENT

The authors declare no conflict of interest.

## Data Availability

All the data of this research work are in the article.

## References

[fsn33913-bib-0001] Arora, S. , & Arora, S. (2021). Nutritional significance and therapeutic potential of Moringa oleifera: The wonder plant. Journal of Food Biochemistry, 45(10), e13933. 34533234 10.1111/jfbc.13933

[fsn33913-bib-0002] Akinwande, B. A. , & Olatunde, S. J. (2015). Comparative evaluation of the mineral profile and other selected components of onion and garlic. International Food Research Journal, 22(1), 332–336.

[fsn33913-bib-0003] Balarajan, Y. , Ramakrishnan, U. , Özaltin, E. , Shankar, A. H. , & Subramanian, S. V. (2011). Anaemia in low‐income and middle‐income countries. The Lancet, 378(9809), 2123–2135. 10.1016/S0140-6736(10)62304-5 21813172

[fsn33913-bib-0004] Belhi, M. , Selmi, H. , Tibaoui, G. , Aloui, F. , Jedidi, S. , & Rouissi, H. (2018). Propriétés chimiques et facteurs antinutritionnels du *Moringa oleifera* . Journal of New Sciences, Agriculture and Biotechnology, CIRS, 11, 3338–3342.

[fsn33913-bib-0005] Bhandari, M. R. , & Kawabata, J. (2006). Cooking effects on oxalate, phytate, trypsin and α‐amylase inhibitors of wild yam tubers of Nepal. Journal of Food Composition and Analysis, 19(6–7), 524–530.

[fsn33913-bib-0006] Blakstad, M. M. , Nevins, J. E. , Venkatramanan, S. , Przybyszewski, E. M. , & Haas, J. D. (2020). Iron status is associated with worker productivity, independent of physical effort in Indian tea estate workers. Applied Physiology, Nutrition, and Metabolism, 45(12), 1360–1367. 10.1139/apnm-2020-0001 32579855

[fsn33913-bib-0007] Broin, M. (2005). Composition nutritionnelle des feuilles de Moringa *oleifer*a. CTA, 5.

[fsn33913-bib-0008] Cappellini, M. D. , Musallam, K. M. , & Taher, A. T. (2020). Iron deficiency anaemia revisited. Journal of Internal Medicine, 287(2), 153–170. 31665543 10.1111/joim.13004

[fsn33913-bib-0009] Cuendet, M. (1999). *Recherche de nouveaux composés capteur de radicaux libres et antioxydants à partir d'une plante d'Indonésie: Fragraea blumei* (*Loganiaceae*) *et de trois plantes d'altitude: Bartsia alpina* (*Scrophulariaceae*), *Loiseleuria procumbens* (*Ericaeae*) *et Campanula ba* . Doctoral dissertation, Université de Lausanne, Faculté des sciences.

[fsn33913-bib-0010] da Silva, I. J. G. , Fonseca, L. , Costa, A. D. S. , & Moita, G. C. (2017). Bioaccessibility and speciation study of iron in bovine tissue. Revista Virtual de Química, 9(2), 476–491.

[fsn33913-bib-0011] Dubock, A. (2017). An overview of agriculture, nutrition and fortification, supplementation and biofortification: Golden Rice as an example for enhancing micronutrient intake. Agriculture & Food Security, 6(1), 1–20.

[fsn33913-bib-0012] Dykes, L. , & Rooney, L. W. (2007). Phenolic compounds in cereal grains and their health benefits. Cereal Foods World, 52(3), 105–111.

[fsn33913-bib-0013] Fernández‐Bedmar, Z. , Demyda‐Peyrás, S. , Merinas‐Amo, T. , & del Río‐Celestino, M. (2019). Nutraceutic potential of two allium species and their distinctive organosulfur compounds: A multi‐assay evaluation. Food, 8(6), 222. 10.3390/foods8060222 PMC6617039 31234398

[fsn33913-bib-0014] Gautam, S. , Platel, K. , & Srinivasan, K. (2010). Higher bioaccessibility of iron and zinc from food grains in the presence of garlic and onion. Journal of Agricultural and Food Chemistry, 58(14), 8426–8429. 20597543 10.1021/jf100716t

[fsn33913-bib-0015] Gibson, R. , & Hötz, C. (2001). Dietary diversification/modification strategies to enhance micronutrient content and bioavailability of diets in developing countries. British Journal of Nutrition, 85, S159–S166. 11509105 10.1079/bjn2001309

[fsn33913-bib-0016] Gibson, R. S. , Bailey, K. B. , Gibbs, M. , & Ferguson, E. L. (2010). Phytate, iron, zinc, and calcium concentrations in plant‐based complementary foods used in low‐income countries and implications for bioavailability. Food and Nutrition Bulletin, 31, 134–146. 10.1177/15648265100312S206 20715598

[fsn33913-bib-0017] Greger, J. L. , & Mulvaney, J. (1985). Absorption and tissue distribution of zinc, iron and copper by rats fed diets containing lactalbumin, soy and supplemental sulfur‐containing amino acids. The Journal of Nutrition, 115(2), 200–210. 4038512 10.1093/jn/115.2.200

[fsn33913-bib-0018] Hurrell, R. F. (2003). Influence of vegetable protein sources on trace element and mineral bioavailability. Journal of Nutrition, 133, S2973–S2977. 10.1093/jn/133.9.2973S 12949395

[fsn33913-bib-0019] Khadija, B. , & Marwa, B. (2021). Effet des solvants d'extraction sur la composition chimique de: Allium cepa et Allium sativum. Université de Biskra.

[fsn33913-bib-0020] Kumar, V. , Sinha, A. K. , Makkar, H. P. S. , & Becker, K. (2010). Dietary roles of phytate and phytase in human nutrition. Food Chemistry, 120, 945–959.

[fsn33913-bib-0021] Lestienne, I. , Icard‐Vernière, C. , Mouquet, C. , Picq, C. , & Trèche, S. (2005). Effects of soaking whole cereal and legume seeds on iron, zinc and phytate contents. Food Chemistry, 89(3), 421–425.

[fsn33913-bib-0022] Liu, J. , Ji, F. , Chen, F. , Guo, W. , Yang, M. , Huang, S. , Zhang, F. , & Liu, Y. (2018). Determination of garlic phenolic compounds using supercritical fluid extraction coupled to supercritical fluid chromatography/tandem mass spectrometry. Journal of Pharmaceutical and Biomedical Analysis, 159, 513–523. 30053709 10.1016/j.jpba.2018.07.020

[fsn33913-bib-0023] Lonnerdal, B. (2010). Calcium and iron absorption‐mechanisms and public health relevance. Internationnal Journal for Vitamin and Nutrition Research, 45, 293–299. 10.1024/0300-9831/a000036 21462112

[fsn33913-bib-0024] Lopez, H. W. , Leenhardt, F. , Coudray, C. , & Remesy, C. (2002). Minerals and phytic acid interactions: Is it a real problem for human nutrition? International Journal of Food Science and Technology, 37, 727–739.

[fsn33913-bib-0025] Macheix, J. J. , Fleuriet, A. , & Jay‐Allemand, C. (2005). Les composés phénoliques des végétaux: un exemple de métabolites secondaires d'importance économique. PPUR presses polytechniques.

[fsn33913-bib-0026] Mawouma, S. , & Mbofung, C. M. (2014). Usages alimentaires de Moringa oleifera dans la région de l'Extrême‐Nord Cameroun. International Journal of Biological and Chemical Sciences, 8(4), 1847–1852.

[fsn33913-bib-0027] Mawouma, S. , Ponka, R. , & Mbofung, C. M. (2017). Acceptability and solubility of iron and zinc contents of modified Moringa oleifera sauces consumed in the far‐north region of Cameroon. Food Science & Nutrition, 5(2), 344–348. 28265369 10.1002/fsn3.398 PMC5332257

[fsn33913-bib-0028] McGee, E. J. T. , & Diosady, L. L. (2018). Prevention of iron‐polyphenol complex formation by chelation in black tea. LWT, 89, 756–762.

[fsn33913-bib-0029] Milman, N. T. (2020). A review of nutrients and compounds, which promote or inhibit intestinal iron absorption: Making a platform for dietary measures that can reduce iron uptake in patients with genetic haemochromatosis. Journal of Nutrition and Metabolism, 2020, 1–15. 10.1155/2020/7373498 PMC7509542 33005455

[fsn33913-bib-0030] Musa, A. , & Ogbadoyi, E. O. (2012). Effect of cooking and sun drying on micronutrients, antinutrients and toxic substances in Corchorus olitorius (jute mallow). Journal of Nutrition and Food Scence, 2(14), 2–9.

[fsn33913-bib-0031] Nchanji, E. B. , Siri, B. , Ngueguim, M. , Kamdem, F. , Butare, L. , Onyango, P. , & Fungo, R. (2020). Lutter contre les carences en micronutriments au Cameroun par la biofortification du haricot commun. CGIAR.

[fsn33913-bib-0032] Neugart, S. , Baldermann, S. , Ngwene, B. , Wesonga, J. , & Schreiner, M. (2017). Indigenous leafy vegetables of eastern Africa—A source of extraordinary secondary plant metabolites. Food Research International, 100, 411–422. 28964364 10.1016/j.foodres.2017.02.014

[fsn33913-bib-0033] Pauwels, J. , van Ranst, E. , Verloo, M. , & Mvondo, Z. E. (1992). *Manuel de laboratoire de pédologie*, *méthodes d'analyses de sols et de plantes; équipement et gestion des stocks de verrerie et de produits chimiques* . Publications agricoles n° 28, A. G. C. D. Bruxelles. Belgique. 180.

[fsn33913-bib-0034] Rocha, G. , Pereira, S. , Antunes‐Sarmento, J. , Flôr‐de‐Lima, F. , Soares, H. , & Guimarães, H. (2021). Early anemia and neonatal morbidity in extremely low birth‐weight preterm infants. The Journal of Maternal‐Fetal & Neonatal Medicine, 34(22), 3697–3703. 31736385 10.1080/14767058.2019.1689948

[fsn33913-bib-0035] Rousseau, S. , Kyomugasho, C. , Celus, M. , Hendrickx, M. E. , & Grauwet, T. (2020). Barriers impairing mineral bioaccessibility and bioavailability in plant‐based foods and the perspectives for food processing. Critical Reviews in Food Science and Nutrition, 60(5), 826–843. 30632768 10.1080/10408398.2018.1552243

[fsn33913-bib-0036] Salawu, K. , Owolarafe, T. A. , Ononamadu, C. J. , Ihegboro, G. O. , Lawal, T. A. , Aminu, M. A. , & Oyekale, A. J. (2021). Phytochemical, nutritional composition and heavy metals content of Allium cepa (onion) and Allium sativum (garlic) from Wudil central market, Kano state, Nigeria. Biokemistry, 33(4), 311–317.

[fsn33913-bib-0037] Schönfeldt, H. C. , & Pretorius, B. (2011). The nutrient content of five traditional south African dark green leafy vegetables—A preliminary study. Journal of Food Composition and Analysis, 24(8), 1141–1146.

[fsn33913-bib-0038] Shi, J. , & Le Maguer, M. (2003). Lycopene in tomatoes: Chemical and physical properties affected by food processing. Critical Reviews in Biotechnology, 20(4), 293–334. 10.1080/07388550091144212 11192026

[fsn33913-bib-0039] Teshome, M. S. , Meskel, D. H. , & Wondafrash, B. (2020). Determinants of anemia among pregnant women attending antenatal care clinic at public health facilities in Kacha Birra District, Southern Ethiopia. Journal of Multidisciplinary Healthcare, 13, 1007–1015. 33061406 10.2147/JMDH.S259882 PMC7522419

[fsn33913-bib-0040] Vermerris, W. (2006). Phenolic compound biochemistry. Springer.

[fsn33913-bib-0041] Welch, R. M. (2002). Breeding strategies for biofortified staple plant foods to reduce micronutrient malnutrition globally. The Journal of Nutrition, 132(3), 495S–499S. 11880578 10.1093/jn/132.3.495S

[fsn33913-bib-0042] Wu, P. , & Chen, X. D. (2021). Validation of in vitro bioaccessibility assays—A key aspect in the rational design of functional foods towards tailored bioavailability. Current Opinion in Food Science, 39, 160–170.

